# Advances in NK cell therapy for brain tumors

**DOI:** 10.1038/s41698-023-00356-1

**Published:** 2023-02-15

**Authors:** Jawad Fares, Zachary B. Davis, Julian S. Rechberger, Stephanie A. Toll, Jonathan D. Schwartz, David J. Daniels, Jeffrey S. Miller, Soumen Khatua

**Affiliations:** 1 Department of Neurological Surgery, Feinberg School of Medicine, Northwestern University, Chicago, IL 60611 USA; 2 Northwestern Medicine Malnati Brain Tumor Institute, Lurie Comprehensive Cancer Center, Feinberg School of Medicine, Northwestern University, Chicago, IL 60611 USA; 3 Department of Medicine, Division of Hematology, Oncology and Transplantation, Masonic Cancer Center, University of Minnesota, Minneapolis, MN 55454 USA; 4 Department of Neurologic Surgery, Mayo Clinic, Rochester, MN 55905 USA; 5 Department of Molecular Pharmacology and Experimental Therapeutics, Mayo Clinic Graduate School of Biomedical Sciences, Rochester, MN 55905 USA; 6 Department of Pediatrics, Division of Hematology/Oncology, Children’s Hospital of Michigan, Detroit, MI 48201 USA; 7 Department of Pediatric Hematology/Oncology, Section of Neuro-Oncology, Mayo Clinic, Rochester, MN 55905 USA

**Keywords:** Drug development, CNS cancer, Cancer immunotherapy, Immunotherapy

## Abstract

Despite advances in treatment regimens that comprise surgery, chemotherapy, and radiation, outcome of many brain tumors remains dismal, more so when they recur. The proximity of brain tumors to delicate neural structures often precludes complete surgical resection. Toxicity and long-term side effects of systemic therapy remain a concern. Novel therapies are warranted. The field of NK cell-based cancer therapy has grown exponentially and currently constitutes a major area of immunotherapy innovation. This provides a new avenue for the treatment of cancerous lesions in the brain. In this review, we explore the mechanisms by which the brain tumor microenvironment suppresses NK cell mediated tumor control, and the methods being used to create NK cell products that subvert immune suppression. We discuss the pre-clinical studies evaluating NK cell-based immunotherapies that target several neuro-malignancies and highlight advances in molecular imaging of NK cells that allow monitoring of NK cell-based therapeutics. We review current and ongoing NK cell based clinical trials in neuro-oncology.

## Introduction

Brain tumors comprise 1.4% of all cancers, whose treatment options and survival outcomes vary depending on the type of brain tumor^1,2^. Therapeutic advances in chemotherapy, radiation, and neurosurgical procedures have increased survival of patients with brain tumors^3^. Yet, malignancies like high-grade gliomas, medulloblastoma, and diffuse intrinsic pontine glioma (DIPG) continue to portend dismal outcomes due to recurrence and/or progression^4,5^.

Despite aggressive treatment regimens that include surgery, radiotherapy, and chemotherapy, many central nervous system (CNS) tumors remain incurable. Intra-tumoral heterogeneity and the ability of the brain tumor cells to evade the immune system and suppress anti-tumoral immunity are some of the important limitations^1,6,7^. Another major limitation to effective therapy is the blood–brain barrier (BBB), which blocks the access of most targeted drugs delivered systemically into tumor sites^8^, and the significant morbidity and concerning long-term side effects caused by current systemic therapeutic regimens. The lack of lymphatic drainage and low abundance of antigen-presenting cells seem to protect brain tumors from the immune system. However, preclinical, and translational studies in the past decade have changed this perception and showed an important role for immune cells in brain tumors^7,9–13^.

Natural killer (NK) cells are the predominant innate lymphocyte subset that mediate anti-tumor response. They are known to play important roles in anti-tumor immunity in various cancers^14,15^, and their efficacy against brain tumors has been shown in preclinical studies^16–21^. NK cell cytotoxicity against cancer cells involves the secretion of perforin, granzyme B^22–24^, and pro-inflammatory cytokines, such as tumor necrosis factor-α and interferon (IFN-γ)^25–27^. As such, NK cell immunotherapy appears as a promising, novel treatment approach for brain tumors^28^.

This review explores the role of NK cells in the brain tumor microenvironment, emphasizes preclinical research with NK cells, examines effects of NK-cell-based therapies in adult and pediatric patients with brain tumors, and discusses tracking the delivery of infused NK cells through molecular neuroimaging. Obstacles that lie ahead of NK cell therapy advancement and future directions in the fight against brain tumors are further explored.

## NK cells in the brain tumor microenvironment

NK cells have been shown to play a fundamental role in brain metastases, meningiomas^29^, glioblastomas^30^, craniopharyngiomas^31^, DIPG^32^, medulloblastoma, and ependymoma^12^. The tumoral niche takes a toll on the functioning of NK cells due to the immunosuppressive factors expressed on or released by cancer cells. Single-cell analysis of the immunosuppressive microenvironment of glioblastoma demonstrated those NK cells that infiltrated into the tumor lesions, expressed higher levels of CXC chemokine receptor 3 (CXCR3) and lower levels of IFNγ^33^.

**Fig. 1:**
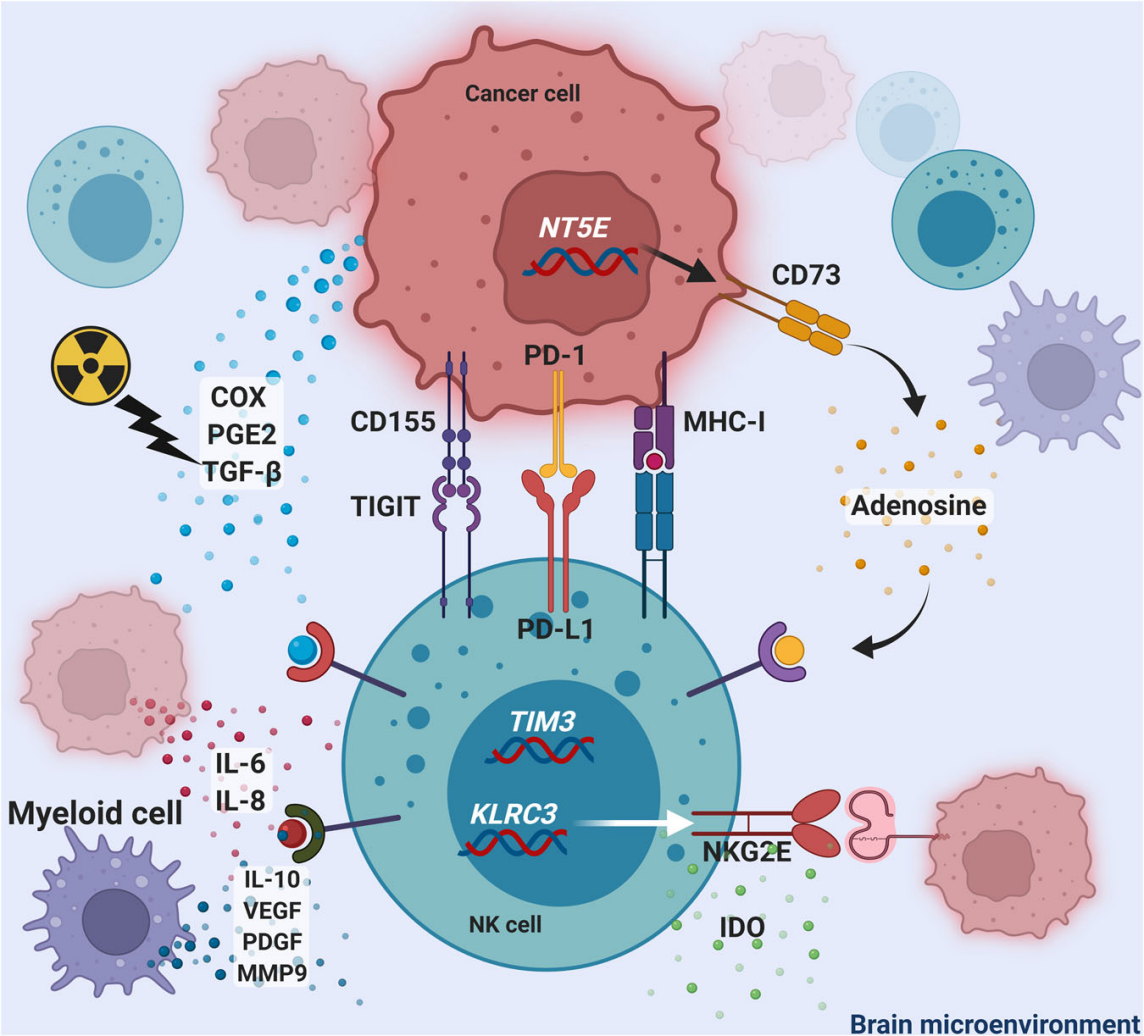
Mechanisms of NK cell deactivation in the brain tumor microenvironment. Mechanisms of NK cell deactivation by cancer cells include the expression of MHC-I, PD-1, CD155, and CD73 expression on cancer cells that can inhibit NK cells. Cytokine and chemokine release, such as IL-6, IL8, IL-10, VEGF, PDGF, MMP9, IDO, adenosine, PGE2, and TGF-beta can further deactivate NK cells. Proteins expressed on NK cells, such as NKG2E and TIGIT, can lead to brain tumor progression.

CNS tumor cells can secrete chemokines, cytokines, and growth factors that diminish the anti-tumor cytotoxicity of NK cells in the brain (Fig. 1). This promotes cancer proliferation, spread, and resistance to treatment. In gliomas, anergic NK cells promote glioma differentiation, which results in the resistance to NK cell-mediated cytotoxicity. Sustained release of interleukin (IL)-6 and IL-8, along with decreased IFN-γ secretion post-differentiation further increases immune resistance to NK cell therapy^34^. Pro-tumoral inflammatory mediators, such as cyclooxygenases (COX) and prostaglandin E2, further weaken NK cell viability and chemokine production^35^.

The NK cell numbers in the brain tumor microenvironment are often depleted, especially after chemo-radiotherapy because of inflammatory mediators like transforming growth factor-β (TGF-β), COX, and prostaglandin E2^35,36^. In a case series of four patients with DIPG, lymphocyte profiling revealed decreased NK cell levels in all four cases^32^. In patients with glioblastoma, the number of NK cells in isolated tumor specimens were significantly decreased after being treated with radiation and temozolomide^36^.

The expression of certain molecules on tumor cells can inhibit NK cell activity. In glioblastoma, major histocompatibility complex class I molecules expressed on tumor cells bind to NK cell inhibitory receptors and suppress their immune function^37,38^. Gliomas and brain metastases can further downregulate the expression of activating receptors, such as NK Group 2 member D (NKG2D), to evade immune activation of host NK cells^39,40^. The tumor microenvironment in pediatric solid tumors is rich in immunosuppressive molecules such as transforming growth factor beta (TGFβ)^41,42^, adenosine^43^, PGE2^44^, as well as indoleamine 2,3-dioxygenase (IDO)^45^. All of these factors have been associated with to significant NK cell dysfunction^46^. As opposed to solid tumors in the adult population, infiltrating and tumor associated macrophages (TAM) are abundantly present in the pediatrics and have been linked to a poor prognosis. Myeloid-derived suppressor cells (MDSC) cells have been also described to commonly infiltrate into the tumor, thereby facilitating the release of various immunosuppressive factors. These include chemokines and cytokines, most of all macrophages-derived IL-10, vascular endothelial growth factor (VEGF), platelet-derived growth factor (PDGF), and matrix metalloproteinase-9, all of which contribute to therapy resistance. In addition, TAMs nurture metastatic spread by upregulating the CCL2/CSF-1 signaling axis in childhood gliomas and portending poor outcomes in neuroblastoma^47,48^. To date, a large body of research has demonstrated that there is a significant number of tumor-infiltrating NK cells that have been associated with good prognosis in many solid tumors^46^. This is reaffirmed in preclinical models where depletion of NK cell populations prior to tumor transplantation induces a more aggressive phenotype with metastatic tumors. These data have now formed a steppingstone to evaluate NK cell-based therapies in brain tumors, which are rapidly gaining attention as an attractive adoptive immunotherapy in pediatrics^49^.

**Fig. 2:**
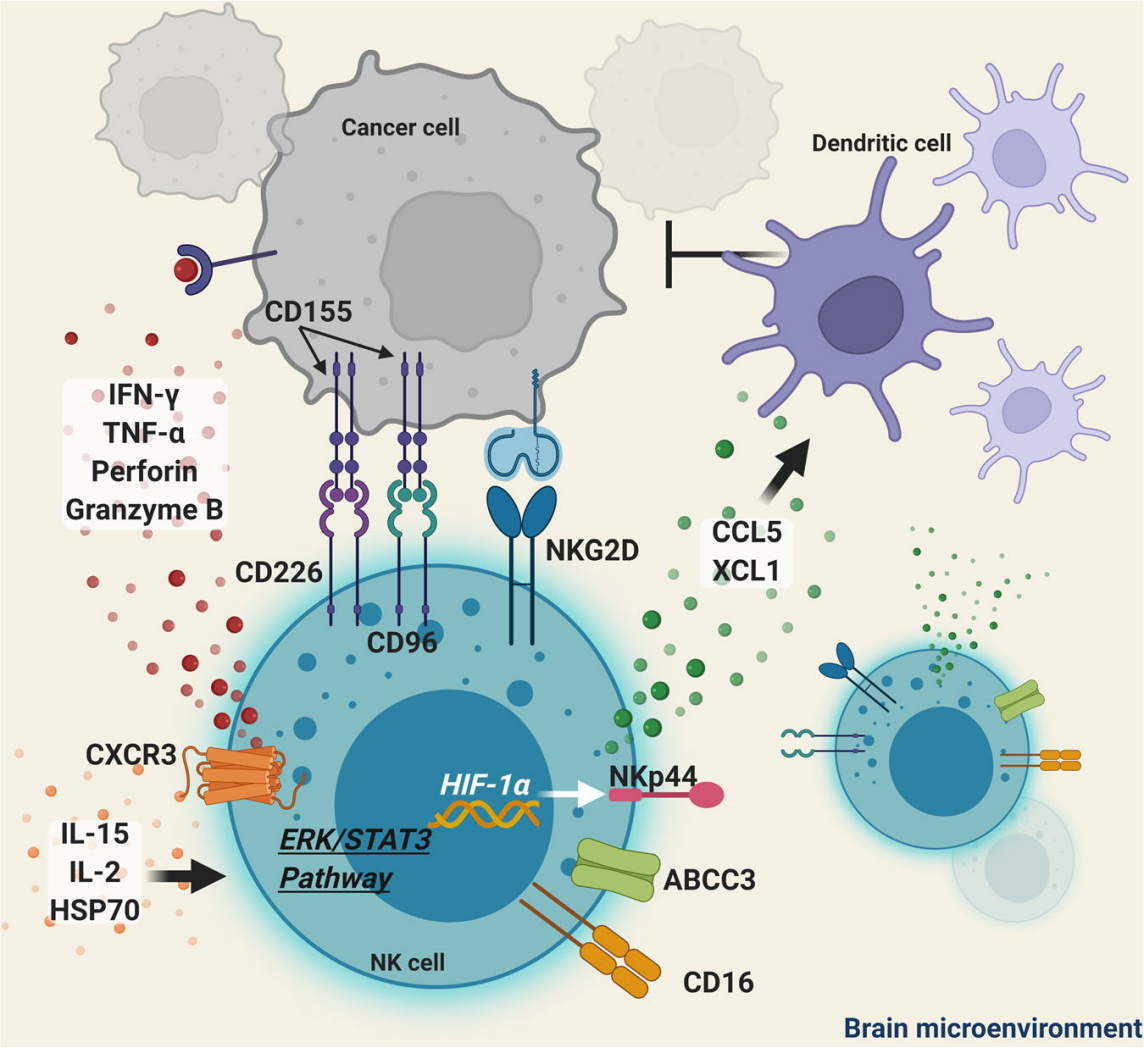
Mechanisms of NK cell activation in the brain tumor microenvironment. Mechanisms of NK cell activation include the secretion of IFN-γ, TNF-α, perforins, and granzyme B. Cytokines, such as IL-2 and IL-15, can also prime the NK cell immune cytotoxic effect. Protein expression of NKG2D, NKp44, CD226, ABCC3, CD16, CXCR3, and CD96 can help NK cells recognize cancer cell antigens and trigger their cytotoxic effect.

Other cytokines and released factors activate NK cells in the brain microenvironment (Fig. 2). IL-15 increases the expression of molecules associated with NK-cell cytotoxicity^50^. IL-2 and heat shock protein 70 (HSP70) improves NK cell homing within the tumor site in vivo^51^, which suggests that the administration of IL-15/HSP70/IL-2-treated NK cells may be a promising therapeutic approach to be considered in the treatment of brain tumors. Increasing NK cell-mediated recruitment of dendritic cells through the release of chemokine ligand 5 (CCL5) and X-C Motif Chemokine Ligand 1 (XCL1) can positively impact survival^35^.

The expression of certain genetic patterns in NK cells or tumor cells can affect brain tumor recurrence and aggressiveness. Silencing of *KLRC3* in NK cells, decreases the self-renewal capacity, invasion, proliferation, radio-resistance, and tumorigenicity of the U87-MG glioma cell line^52^. CRISPR-Cas9-mediated knockout of T cell immunoglobulin mucin family member 3 (TIM3), a marker of dysfunctional NK cells^53,54^, in human NK cells inhibits glioma cellular growth^53^. The expression of CD73 or CD155 in glioma suppresses NK cell presence in the tumor microenvironment and contributes to increased tumor migration and aggressiveness^55,56^. NK cells that express CD16, predominate in glioblastoma patients surviving more than 12 months after surgery without disease progression^57^. This subtype of NK cells co-expresses increased levels of ATP Binding Cassette Subfamily C Member 3 (ABCC3) and IFN-γ, which are related to a strong, long-term NK cell cytotoxic response^57^. NK cells can further induce antibody-dependent cell-mediated cytotoxicity (ADCC) to provide anti-tumor cytotoxicity^58^. This process is mediated through the CD16 receptor on NK cells that binds the Fc portion of IgG antibodies triggering the lysis of tumor cells. The expression of CD1d, an antigen-presenting molecule for NK cells, is common in human glioblastoma but not in glioma stem cells^59^. Retinoic acid upregulates CD1d expression in glioma stem cells in vitro, sensitizing them to NK-mediated cytotoxicity^59^.

Hypoxic stress in the tumor microenvironment can lead to immunosuppressed NK cell phenotypes^60^. Pre-activation of NK cells through exposure to normoxic culture followed by hypoxic conditions allow NK cells to shift metabolically from oxidative phosphorylation into glycolysis^61^. This shift helps NK cells overcome hypoxia-mediated functional impairment. Hypoxia-inducible factor 1-alpha (HIF-1α) activation can upregulate the expression of the natural cytotoxicity receptor NKp44 receptors to reverse impairment in NK cell survival, proliferation, and tumor cytotoxicity^61^. The activation of extracellular signal-regulated kinase (ERK)/ signal transducer and activator of transcription 3 (STAT3) pathways can further expand functionally robust NK cells for adoptive cellular therapy by reducing p21/p53 apoptotic pathways, upregulating cell cycle-promoting genes, *CCNE1*, *CDC6*, *CDC20* and downregulating of cell cycle-arrest genes, *CDKN1A*, *GADD45A*, and *MDM2*^61^.

## Preclinical advancements in NK cell therapy

**Fig. 3:**
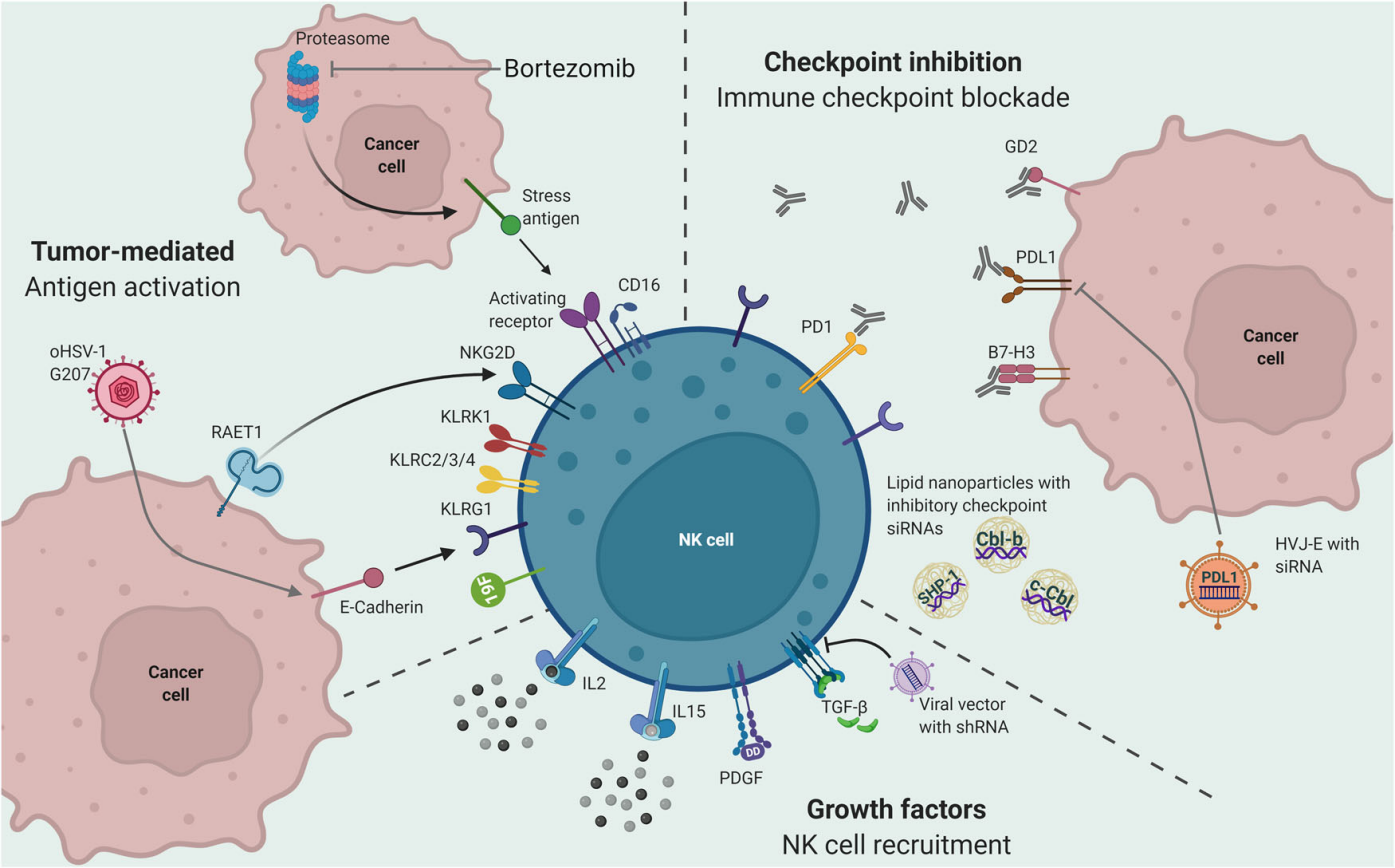
Preclinical approaches of NK cell therapeutics in brain tumors. Preclinical approaches involving NK cells for the treatment of brain tumors focus on three modes of action. Tumor-mediated antigen activation includes the activation of NK cell activity by cancer cell antigen recognition. Recognition of stress antigens, expressed as a result of bortezomib treatment, can activate NK cell receptors. Expression of E-cadherin, primed by oHSV-1 G207, enhances NK cell recognition and activation of NK cells through the KLRG1 receptor. The recognition of the RAET1 antigen on cancer cells by the NKG2D receptor on NK cells can also lead to NK cell activation and cytotoxicity. Other receptors on NK cells, such as KLRK1, and KLRC2/3/4 have been reported to play a role in NK cell activation against cancer cells in the brain tumor microenvironment. Growth factors, such as IL2, IL15, and PDGFDD, promote NK cell recruitment and cytotoxicity. Inhibition of TGF-beta, using viral vectors carrying shRNA, has been shown to increase NK cell activation against brain tumor cancer cells in preclinical settings. Checkpoint inhibition and immune checkpoint blockade through antibodies against GD2, PD-L1, PD-1, and B7-H3, has been reported to enhance NK cell activity in brain tumors. Viral vectors carrying siRNA targeting PD-L1 on cancer cells or lipid nanoparticles carrying siRNAs against inhibitory checkpoints, such as Cbl-b, SHP-1, and c-Cbl, on NK cells, have also been reported to activate the NK cell immune response against brain tumor cells.

Novel immunotherapeutic approaches and cytotoxic agents against brain tumor cells can promote NK cell immune response (Fig. 3). Ex vivo expansion of NK cells and their re-introduction into tumor setting has been tested in preclinical settings. NK cells that have been expanded ex vivo possess the cytolytic ability to target medulloblastoma cells in vitro^62,63^. Retro-orbital administration of ex vivo expanded and activated NK cells along with IL-2 prolonged the survival of NOG mice bearing subcutaneous U87MG-derived tumors^64^. The combination of ex vivo expanded NK cells with an IL-15 agonist and dinutuximab, an anti-disialoganglioside GD2 monoclonal antibody, significantly increases cytotoxicity against pediatric glioma cells and improves survival in preclinical models^65^. Interim analysis of a phase 1 clinical trial using anti-GD2 CAR NK cells, showed that the cells underwent in vivo expansion and localization to the tumor site in patients with relapsed or refractory neuroblastoma (Table 1).

**Table 1:** Preclinical approaches in NK cell therapeutics for brain tumors.

Cell type	Malignancy	Treatment	Outcome	Ref
NK-mAb9.2.27	Glioblastoma	Administered to athymic rats via convection enhanced delivery	Tumors regressed on MRI 3 months post treatment. Therapy promoted the recruitment of macrophage and microglia with a pro-inflammatory phenotype	^18^
HSP70/IL-2 Treated NK cells	Glioblastoma	NK Cells were intracranially and systemically injected into male rats with C6 GBM expanded cell lines	IL-2/IL-15 treated NK cells were cytotoxic in vitro. Tumor size decreased	^207^
PBMC-derived NK cells	Glioblastoma	U251MG and U343MG cell lines were treated with N6-isopentenyladenosine (iPA) for 24 h to assess cell cycle, proliferation, and NK cell cytotoxicity	iPA enhances NK cell-mediated lysis of glioblastoma cells via p53 activation. In vivo in U87-xenograft mice NKG2D ligands were upregulated by iPA	^208^
PBMC-derived NK cells	Glioblastoma	U87MG, T98G, and LN-18 cell lines were incubated with NK cells to assess cytotoxicity. Growth inhibition was assessed with NK cells alone versus NK cells and temozolomide	NK cells and temozolomide therapy significantly decreased glioma cell viability. Apoptosis was induced after 24 h, regardless of temozolomide therapy	^74^
Mouse-derived NK cells from C57BL/6 mouse spleens	Glioblastoma	C57BL/6 female mice were intracranially injected with either: (1) NK cells and GL261 GSCs, (2) NK cells and anti-B7H1 GSCs, (3) anti-PD1 NK cells and GSCs, or (4) anti-PD1 NK cells and anti-B7H1 GSCs.	In vitro, lysis of GL261 GSCs was significantly higher in the double inhibited group compared to other groups. In vivo, anti-PD1 NK cells significantly inhibited GL261 GSCs tumor growth compared to the other groups.	^67^
PBMC-derived IL-15 induced NK cells	Glioblastoma	Patient-derived GSCs were used in orthotopic xenograft mouse models. Cytotoxic assays were performed.	Tumor cells differentiated from GSCs were more sensitive to NK cell mediated killing than GSCs themselves. TGF-b family cytokines played a key role in glioma proliferation, invasion, and immunosuppression.	^68^
PBMC-derived IL-2 and IL-15 induced NK cells	Glioblastoma	P3, 2012–018, BG8 and BG9 patient-derived tumor lines were established from glioblastoma tissue and assigned to 4 groups: (1) Control, (2) Bortezomib, (3) NK cells, and (4) BTZ + NK cells. NOD/SCID mice implanted with BG7 tumor were subjected to one of the treatment groups.	NK cell lysis killed at increasing effector: target ratios, at 24-46% at an E:T ratio of 5:1. In vitro, the combination treatment was more effective than monotherapy. In mice, autologous NK cells alone or in combination with Bortezomib diminished tumor volume and prolonged survival.	^84^
PBMC-derived NK cells	Glioblastoma	Cytotoxicity assays were performed with NK cells cultured for 2 weeks against glioblastoma cells. In vivo NK cell therapy was administered intracranially into NOD/SCID mice with P3 tumors.	Donor NK cells efficiently killed cells from both P3 and 2012-018 cell lines. Blocking NKG2D receptor attenuated NK cell-mediated killing. In vivo, mice treated with a single dose of NK cells lived significantly longer than untreated controls. More than one dose diminished this survival benefit.	^140^
PBMC-derived NK cells	Glioblastoma	The impact of bortezomib combined with oncolytic HSV on tumor cell death and sensitivity to NK cell immunotherapy was assessed.	Combination treatment induced necroptotic cell death and significantly enhanced NK cell activation and improved anti-tumor efficacy.	^209^
Umbilical cord blood-derived NK cells	Medulloblastoma	Cytokine secretion of PG13 cell line expressing TGF-b double negative receptor II (TGF-b DNRII) expressing NK cells were measured after supernatants were collected and a second stimulation was performed.	High levels of suppressive TGF-b were found in medulloblastoma cells. Umbilical cord blood-derived NK cells modified to express TGF-b DNRII can protect against exogenous TGF-b and medulloblastoma-mediated immune suppression.	^72^
PBMC-derived NK cells	Medulloblastoma	Immunohistochemistry from 54 tumors were analyzed and level of MHC class I related chains A (MICA) and UL16 binding protein (ULPB-2) was assessed.	46% of the tumors demonstrated strong MICA expression and the percentage of ULPB-2 immunoreactivity was <25%. Compared to erythroleukemia and neuroblastoma cell lines, medulloblastoma was the most resistant to resting NK cell cytotoxicity. This improved twofold when NK cells were stimulated with IL-15.	^63^
PBMC-derived NK cells	Medulloblastoma	NK cell-mediated lysis of 1603-MED, HTB-186, and HTB-185 cell lines was assessed using ^51^Cr-relase assay. Cell lines were analyzed for surface expression of molecules exhibiting stem cell phenotypic properties.	Highly purified, activated NK cells efficiently kill different cell lines with the NK receptors involved in lysis differing depending on the cell line. HTB-185 and 1603-MED expressed CD133, while all cell lines expressed CD146 and B7-H3. Cytoplasmic NB84 was expressed by HTB-186 and HTB-185.	^130^

Anti-tumor immunity in the brain is restrained by multiple tumor-induced immunosuppressive mechanisms. Targeting immunosuppression may unleash NK cell anti-tumor cytotoxicity in the brain. Programmed death 1 (PD-1)/PD-L1 inhibitors can potentiate the NK cell response against brain tumors, such as gliomas^66,67^. As NK cell inhibition is usually accompanied by the expression of multiple immune checkpoint molecules on T cells^68^, therapies involving combinations of immunotherapeutic agents and NK cells can be vital in overcoming therapeutic resistance. NK cells that are platelet-derived growth factor D (PDGF-DD) activated and/or highly express killer cell lectin-like receptors (KLR), such as KLRK1 and KLRC2, KLRC3 and/or KLRC4, are associated with better prognosis in low-grade glioma^69^. Using a lipid nano-carrier encapsulating small interfering RNAs that gene silence the key intrinsic inhibitory NK cell molecules, SHP-1, Cbl-b, and c-Cbl, led to increased NK cell activation, and tumor killing^70^. Labeling NK cells with a fluorine-19 (19F)-based perfluorocarbon emulsion enhanced the suppression of medulloblastoma growth in mouse orthotopic models while enabling 19F MRI to provide feedback on the delivery of infused NK cells^71^. TGFβ neutralization sensitize medulloblastoma cells to NK cell cytotoxicity in vitro^72^. Targeting the αv integrin/TGF-β axis further improves NK cell function against glioblastoma stem cells^73^. Treatment of glioma stem cell-engrafted mice with allogeneic NK cells in combination with inhibitors of integrin or TGF-β signaling or with TGFBR2 gene-edited allogeneic NK cells prevented glioma-induced NK cell dysfunction and tumor growth^73^. In glioma, suboptimal doses of N6-isopentenyladenosine upregulate cell surface expression of NKG2D ligands, which facilitates NK cell-mediated cytotoxicity^74^. The combination of NK cells and temozolomide enhances temozolomide-induced inhibition and apoptosis in U87MG glioma cell lines and overcomes temozolomide resistance^74^. Resveratrol and IL-2 enhance the cytolytic activity of NK cells by upregulating the expression of c-Myb, a downstream transcription factor of Akt and mTORC2^75^. The administration of resveratrol and IL-2 in humans and mice increased NK cell activity in the blood and effectively inhibited tumor growth and metastasis in mice^76^. Using hemagglutinating virus of Japan-envelope (HVJ-E) a non-replicating virus-derived vector, it was possible to deliver siRNA that target PD-L1 expression^77^. HVJ-E provoked a robust anti-tumoral immunity by activating NK cells and CD8 + T lymphocytes^77^.

Oncolytic viral therapy for brain tumors suffers from poor intra-tumoral viral spread and rapid clearance by NK cells. Oncolytic Herpes Simplex Virus (oHSV) engineered to encode E-cadherin, a ligand for NK cell inhibitory receptor KLRG1, protects virus-infected cells from NK cell killing, thereby enhancing viral spread, facilitating cell-to-cell infection and viral entry, and reducing viral clearance^78^. Removing the genes that are essential for viral replication in normal brain tissue prevents productive infection of normal cells while permitting conditional replication in tumor cells. oHSV expressing anti(α)-human CD47 IgG1 or IgG4 antibody were capable of blocking CD47, an immune checkpoint in glioblastoma cells, thereby decreasing local tumor size and improving survival in murine models of glioblastoma^79^. NK cells, along with macrophages, mediated the anti-tumor cytotoxic response and the antibody production by the oHSV^79^. The engineered oHSV-1 G207 oncolytic virus, which selectively replicates in brain tumor cells^80,81^, sensitized pediatric high-grade gliomas to virotherapy in preclinical models^13^. The combination of oncolytic viral therapy with NK cell therapy can also result in a synergistic, therapeutic effect against brain tumors. The combination of bortezomib, a proteasome inhibitor, oHSV, and NK cell therapy, demonstrated synergetic effects and therapeutic efficacy., Treatment of oHSV-infected gliomas with bortezomib induced necroptotic cell death to enhance NK-cell immunotherapy^82,83^. Bortezomib treatment of cancer cells induces stress antigens that are identified by NK-activating receptors^84^. This leads to the release of cytolytic molecules, such as IFN-γ, perforin, and granzyme A by NK cells, which disrupts mitochondrial function and leads to glioblastoma cell death^84^.

In recent years, efforts have been made to provide NK cells with a means of responding to specific cell expressed antigens. This has been achieved through transduction of primary NK or NK cell lines with chimeric antigen receptors (CAR)^85,86^. CARs consist of an antigen-specific extracellular domain, a transmembrane domain and an intracellular signaling domain that utilizes down-stream signaling adapters such as the CD16 signaling molecules, CD3ζ or FcεR1γ^87^. One major benefit of using NK cells is that they do not cause graft versus host disease in the allogeneic setting^88^, making them a safe choice for an off-the-shelf product. Activating cytokines, such as IL-2 or IL-15, may also aid in the persistence, expansion, and trafficking of CAR-NK cells. To date, several CAR-T therapies have been evaluated clinically. These include EGFRvIII, IL13Ra2, and HER2. In the EGFRvIII trial no adverse events were observed up to a dose of 1 × 10^10^ cells. However, two patients receiving a dose of 3 × 10^10^ cells resulted in serious adverse events including dyspnea and hypoxia while another patient receiving 6 × 10^10^ cells died 4 h after administration^89^. Median progression-free survival in this trial was 1.3 months with an overall median survival of 6.9. Additionally, all patients developed hematologic toxicities from the preparative chemotherapy. The current methodology to generate CAR-T cells requires leukapheresis to acquire the patient’s own T cells to activate and transduce with the desired CAR construct, which then must be expanded and selected for the CAR expressing T cells. This process is lengthy and expensive and leaves the possibility of adverse events including cytokine release syndrome (CRS). Alternatively, NK CAR products can be derived from haploidentical, patient-derived, ex vivo expanded NK^90^, NK cell lines such as NK92^91–93^ or from iPSC-derived NK cell products^94,95^ constituting an off-the-shelf product that can be administered regardless of patient MHC background and have demonstrated limited adverse effects compared to CAR-T therapies^96^. Thus, NK cells represent a robust and flexible platform for a variety of CARs that may target CNS tumor-specific antigens, with less neurotoxicity and cytokine release syndrome seen with CAR-T cell products.

Currently, there are more than 30 ongoing clinical trials using CAR-NK derived from a variety of sources including NK cell-lines, primary peripheral blood NK, umbilical cord blood-derived NK, hematopoietic stem cell (HSC) derived NK^97^, and iPSC-derived NK targeting a variety of liquid and solid tumors. However, a major challenge to using HSC-derived NK cells is the limited expansion capacity of the initial pool of primary cells while still preserving their stem-cell-like properties. NK cell lines are an attractive NK cell source as, unlike primary cell sources, they require no purification from contaminating cell populations, can be grown in large numbers and are easily transducible^98^. While there is limited clinical data for CAR-NK products, there are more than 100 pre-clinical studies (www.carnkreview.com) demonstrating good in vitro and in vivo efficacy in animal models^87^ (Table 2).

**Table 2:** Preclinical approaches with CAR-NK cell therapeutics for brain tumors.

Target	Cell type	Malignancy	Treatment	Outcome	Ref
ErbB2 (HER2)	NK-92	Breast cancer brain metastases	Orthotopic xenografts in athymic nude rats. Administered by I.V. injection.	Early intensive treatment with targeted NK-92 cells and ultrasound improved survival compared with biweekly treatments or either treatment alone. The intensive treatment paradigm resulted in long-term survival in 50% of subjects.	^210^
EGFR	NK-92, PB-NK	Breast cancer brain metastases	Orthotopic xenografts in NSG mice. Administered by intratumoral injection.	EGFR-CAR-engineered NK-92 cells and primary NK cells displayed enhanced cytotoxicity and IFN-γ production when co-cultured with breast cancer cell lines MDA-MB-231, MDA-MB-468, and MCF-7. Intratumoral administration of either EGFR-CAR-transduced NK-92 cells or oHSV-1 mitigated tumor growth.	^106^
GD2	NK-92	Neuroblastoma	In vitro only.	Enhanced cell killing was strictly dependent on specific recognition of the target antigen and could be blocked by GD2-specific antibody or anti-idiotypic antibody occupying the CAR’s cell recognition domain.	^211^
EGFR and EGFRvIII	NK-92	Glioblastoma	Orthotopic xenografts in NSG mice. Administered by intratumoral injection.	In vitro analysis revealed high and specific cytotoxicity of EGFR-targeted NK-92 against established and primary human GBM cells, which was dependent on EGFR expression and CAR signaling. In immunodeficient mice carrying intracranial GBM xenografts local treatment with dual-specific NK cells was superior to treatment with the corresponding monospecific CAR NK cells. This resulted in a marked extension of survival.	^104^
EGFR and EGFRvIII	NKL	Glioblastoma	Orthotopic xenografts in NSG mice. Administered by intratumoral injection.	EGFR-CAR-engineered NK cells displayed enhanced cytolytic capability and IFN-γ production when co-cultured with glioblastoma cells or patient-derived glioblastoma stem cells in an EGFR-dependent manner. Intracranial administration of NK-92-EGFR-CAR cells resulted in efficient suppression of tumor growth and significantly prolonged the tumor-bearing mice survival.	^85^
GD2	NK-92-MI	Neuroblastoma	Orthotopic xenografts in NSG mice. Administered by intratumoral injection.	CAR NK-92 cells induced specific killing of GD2-expressing cells in vitro and in vivo, associated with enhanced production of interferon-γ.	^91^
EGFRvIII	YTS	Glioblastoma	Subcutanous xenografts in NMRI nude mice. Administered by intravenous injection.	EGFRvIII-CAR confers specific cytotoxicity of NK cell towards EGFRvIII+ glioblastoma cells in vitro and to established subcutaneous U87-MGEGFRvIII tumor xenografts.	^212^
EGFRvIII	KHYG-1	Glioblastoma	In vitro only.	EGFR-CAR-KHYG-1 inhibited GBM cell-growth via apoptosis in an EGFRvIII-expressing specific manner.	^213^
EGFRvIII	KHYG-1	Glioblastoma	Subcutaneous xenografts in NOD/SCID mice. Administered by subcutaneous injection.	EGFR-CAR-KHYG-1 cells led to expression of cellular immunity-related cytokines on EGFRvIII-expressing U87MG in vitro but did not inhibit tumor progression in vivo.	^214^
GD2	NK-92	Neuroblastoma	Subcutaneous xenografts in NSG mice. Administered by peritumoral injection.	NK-92-scFv(ch14.18)-zeta to effectively lyse GD2( + ) neuroblastoma cells also including partially or multidrug-resistant lines. NK-92-scFv(ch14.18)-zeta is able to mediate a significant anti-tumor response in vivo in a drug-resistant GD2( + ) neuroblastoma xenograft mouse model.	^215^
ErbB2 (HER2)	NK-92	Glioblastoma	Orthotopic xenografts in NSG mice. Administered by intratumoral injection.	Potent in vivo anti-tumor activity of NK-92/5.28.z (GD2) was observed in orthotopic GBM xenograft models in NSG mice, leading to a marked extension of symptom-free survival upon repeated stereotactic injection of CAR NK cells into the tumor area. Local therapy with NK-92/5.28.z cells resulted in cures of transplanted syngeneic GBM in four of five mice carrying subcutaneous tumors and five of eight mice carrying intracranial tumors.	^105^
GD2	PB-NK	Neuroblastoma	In vitro only.	Genetic modification with 14.G2a-2B4ζ significantly enhanced the activation response of NK cells to the neuroblastoma cell line JF. JF cells were susceptible to lysis by two of three unmodified NK cell cultures, but resistance to the third NK cell line was successfully overcome by the signal-enhanced receptor.	^216^

**Fig. 4:**
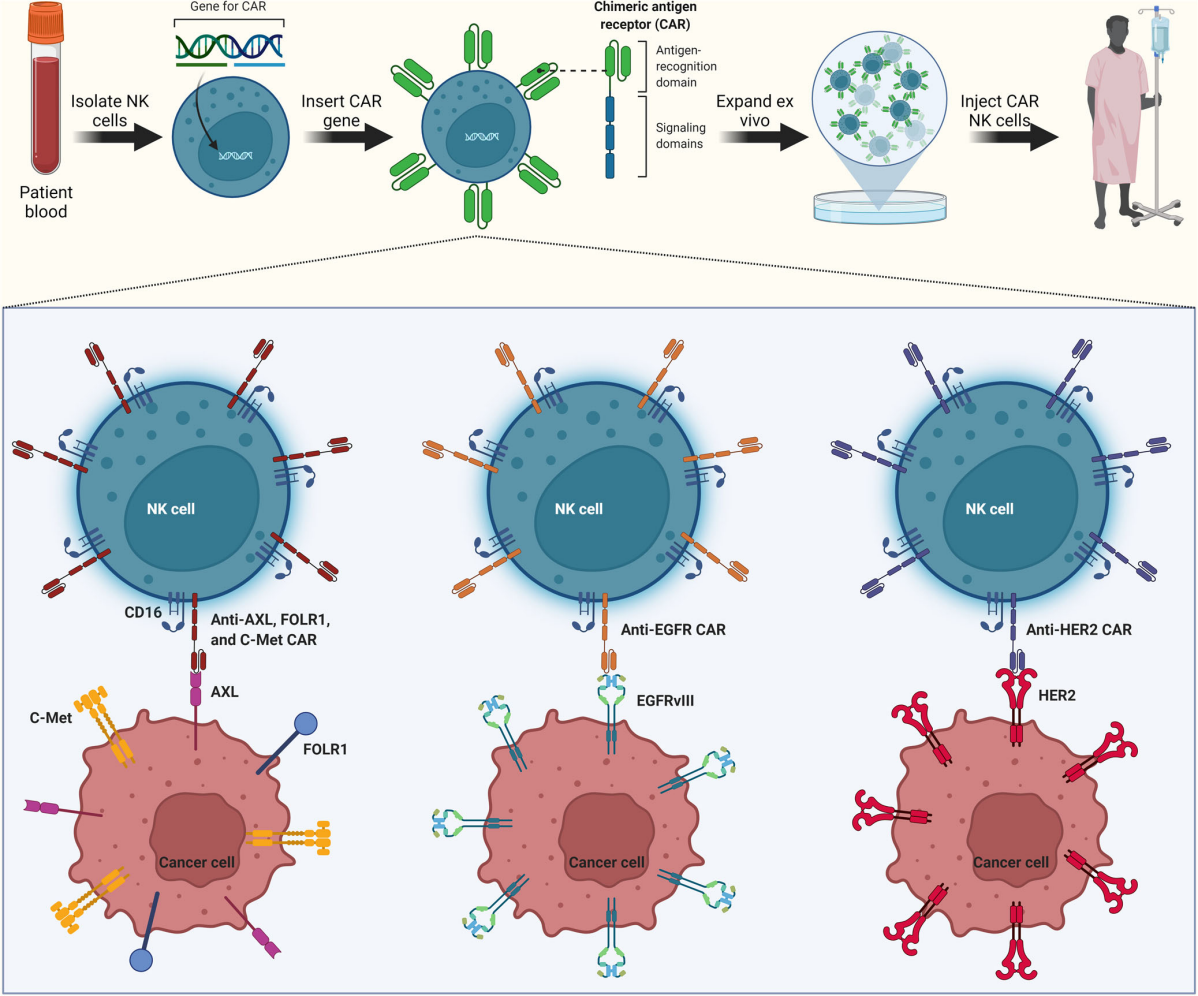
Adoptive CAR NK cell therapeutics in brain tumors. Chimeric antigen receptor (CAR) approaches utilize primary NK cells (isolated from patients) or NK cell lines and their transduction with a CAR gene to produce CARs. CARs consist of an antigen-recognition domain and signaling domain that utilizes down-stream signaling adapters such as the CD16 molecule. Following expansion of the CAR NK cells ex vivo, the cells are injected back into patients with brain tumors. In vitro, CAR KHYG-1 NK cells have been used to target c-Met, FOLR1, and AXL receptors that are overexpressed in glioblastoma cell lines. In preclinical settings, anti-EGFR/EGFRvIII and anti-HER2 CAR-NK cells have also demonstrated enhanced cytotoxicity against glioblastoma cells in vitro and resulted in increased tumor control and survival in animal models. Anti-EGFR CAR NK cells further showed lytic effects in combination with oncolytic virotherapy in metastatic brain cancer.

In vitro studies using CAR-NK to target glioblastoma have focused on CAR-targeting of upregulated proteins in brain tumor cells (Fig. 4). CAR KHYG-1 NK cells that can target c-Met also known as hepatocyte growth factor receptor (HGFR) and AXL proteins that are overexpressed in glioblastoma cell lines, led to cytokine secretion and glioblastoma cell lysis^99^. Epidermal growth factor receptor (EGFR) and it’s variant, EGFRvIII, are well-established therapeutic targets in glioblastoma^100^ expressed in 40–60% of glioblastoma tumors^101^ and minimal expression on surrounding tissue^102^. Similarly, human epidermal growth factor receptor 2 (HER2), a member of the family of EGFR-related receptor tyrosine kinases, is expressed in nearly 80% of glioblastoma tumors^103^. Preclinical trials with both EGFR/EGFRvIII^104^ and HER2^105^ CAR-NK have demonstrated enhanced cytotoxicity against glioblastoma cells in vitro and resulted in increased tumor control and survival in animal models. Use of a HER2 CAR-NK in an orthotopic xenograft glioblastoma mouse model inhibited tumor progression and significantly extended survival^105^. Furthermore, mice that were re-challenged in the other brain hemisphere rejected the newly implanted tumor more than 120 days after the initial therapy^105^. In metastatic brain cancer, CAR NK cells that overexpress EGFR in combination with oHSV-1 showed a synergistic lytic effect as compared to monotherapy.^106^ The combination therapy increased IFN-γ production and significantly improved survival.^106^ The administration of oHSV that release IL15, a cytokine that activates the immune system, along with EGFR-CAR NK cells improved the survival of NK and CD8 T cells^107^. This, in turn, inhibited tumor growth and prolonged survival of glioblastoma-bearing mice^107^. The combination of CAR NK cells designed to target human leukocyte antigen G (HLA-G), an immune checkpoint protein, with low-dose chemotherapy effectively reduced xenograft brain tumor growth and extended median survival in orthotopic mouse models^108^. This suggested that the reversal of the HLA-G-mediated immunosuppression restores native NK cytolytic functions. CAR NK cells designed to activate the glioblastoma immune-metabolic microenvironment by inhibiting CD73, suppressing adenosine production, and inhibiting autophagy have decreased tumor size in preclinical models using patient-derived xenografts^109^.

## NK cell therapy for adult brain tumors

Human studies on brain tumors provide evidence to support the use of NK cells for therapy. In patients with glioblastoma, the presence of activated, CD16-positive NK cells were significantly associated with improved survival^110^. The decrease in levels of activated NK cells correlated to transition from low to high-grade brain tumors, as higher levels of activated NK cells were found in low-grade gliomas than in high-grade gliomas^111^ (Table 3).

**Table 3:** Clinical trials using NK cell therapies for adult brain tumors.

NCTID	Trial	Phase	Number enrolled	Inclusion criteria	Outcome	Ref
–	Autologous Natural Killer Cell Therapy for Human Recurrent Malignant Glioma	–	9	Patients with recurrent malignant gliomas	Intravenous injection of autologous NK cells and low-dose interferon beta was performed. 3/9 patients had partial response and 2/9 had mild response to treatment. Severe neurological toxicity was not observed in any of the patients.	^112^
KCT0003815	Autologous adoptive immune-cell therapy elicited a durable response with enhanced immune reaction signatures in patients with recurrent glioblastoma	I/IIa	14	Patients with recurrent glioblastoma	No severe adverse events were observed. Median overall survival was 22.5 months, and the median progression-free survival was 10 months. Five patients were alive for over 2 years and showed durable response with enhanced immune reaction transcriptomic signatures without clinical decline.	^113^
–	Audencel Immunotherapy Based on Dendritic Cells Has No Effect on Overall and Progression-Free Survival in Newly Diagnosed Glioblastoma	II	76	Patients with newly diagnosed glioblastoma	Administering autologous dendritic cells that have been charged with the tumor lysate for an average of seven months did not show toxicity attributable to vaccination. Progression-free survival at 12 months did not differ significantly between the control and vaccine groups (28.4% versus 24.5%). Median overall survival was similar with 18.3 months for both groups.	^118^
NCT01588769	Tolerability and Efficacy of ALECSAT Administered to Glioblastoma Multiforme Patients	I	25	Patients with recurrent glioblastoma	Cytotoxic lymphocytes, including NK cells, generated ex vivo by exposure to antigens induced by DNA demethylation homed to the tumor, with tumor regression ongoing in three patients for 14, 22, and 27 months, respectively. No treatment-related adverse effects were observed.	^120^
NCT03383978	Intracranial Injection of NK-92/5.28.z Cells in Patients With Recurrent HER2-positive Glioblastoma	I	30	Patients with recurrent HER2+ glioblastoma	No outcomes yet.	–
NCT02839954	CAR-pNK Cell Immunotherapy in MUC1 Positive Relapsed or Refractory Solid Tumor	I/II	10	Patients with recurrent glioma	No outcomes yet.	–

NK cell therapeutics have mostly focused on overcoming the immunosuppressive signals from the tumor microenvironment and amplifying the effect of NK cells with combination therapy. Adoptive immunotherapy using NK cells that have been expanded and activated ex vivo have made it into clinical trials for patients with high-grade gliomas. Administration of autologous NK cells to patients with recurrent malignant gliomas has been shown to be safe and partially effective; four of nine patients recorded tumor regression^112^. In a phase I/IIa clinical trial, adoptive, ex-vivo-expanded, and activated NK cells and T lymphocytes from peripheral blood mononuclear cells of patients with recurrent glioblastoma was safe, with a median overall survival of 22.5 months and a median progression-free survival of 10 months^113^. Five of 14 patients (36%) showed a durable response and were alive for over two years^113^. The combination of NK cell therapy with mTOR inhibitors was safe in adult patients with brain tumors^114^. At the 6th month interval, disease control rate was 72%^114^. The combination of monalizumab, a humanized anti-NKG2A antibody, and cetuximab, an EGFR inhibitor, was effective in promoting ADCC in patients with head and neck squamous cell carcinoma^115^. A phase II trial utilizing this combination therapy showed a 31% objective response rate^115^. As EGFR is a prime target for gliomas and contribute to temozolomide resistance^116,117^, the combination therapy can be a viable option for glioma treatment and induction of NK cell anti-tumor activity.

Cancer vaccines designed to prime an anti-tumor, cytotoxic response against using antigen-presenting cells, can employ NK cells as effector cells. In adult patients with newly diagnosed glioblastoma, administering autologous dendritic cells that have been charged with the tumor lysate for an average of seven months was safe but clinically ineffective^118^. Nevertheless, the vaccine was capable of significantly increasing the levels of IFN-γ, the transcription factor T-bet in the blood, and IL-2 in a dose-dependent manner^119^. Levels of IFN-γ and CD8 + NK cells in the blood after vaccination correlated significantly with better survival^119^. The usage of CD4 + T helper cells, activated by DNA-demethylating agents, as antigen-presenting cells can also generate autologous cytotoxic T lymphocytes and NK cells^120^. Treatment of three of 25 adult patients with recurrent glioblastoma led to tumor regression for up to 27 months, with no treatment-related adverse events^120^.

Clinical trials with CAR NK-cell-based therapeutics are currently ongoing. The intracranial injection of NK-92/5.28.z CAR NK cells in patients with recurrent HER2-positive glioblastoma is being evaluated for safety and tolerability to determine the maximum tolerated dose for a phase 2 trial (NCT03383978). A trial evaluating CAR-NK cell immunotherapy in relapsed or refractory solid tumors, including malignant gliomas, is enrolling patients to receive a novel specific CAR NK cell targeting the MUC1 antigen (NCT02839954).

## NK cell therapy for pediatric brain tumors

Brain tumors in the pediatric population harbor reduced NK cell-mediated immune surveillance and are linked to a less immunosuppressive tumor microenvironment compared to their adult counterparts. Furthermore, the phenotypic characteristics of the pediatric brain tumor microenvironment differ between molecular and histological tumor types^121^. This emphasizes the importance of a tailored therapeutic approach with immunotherapy against pediatric brain tumors, which should also consider the phenotype of the tumor microenvironment (inhibited immune surveillance) and its complex interaction with the bulk tumor (Table 4).

**Table 4:** Clinical trials using NK cell therapies for pediatric brain tumors.

NCTID	Trial	Phase	Number enrolled	Inclusion criteria	Outcome	Ref
NCT02271711	Expanded Natural Killer Cell Infusion in Treating Younger Patients With Recurrent/Refractory Brain Tumors	I	12 (9 received therapy)	Age <21 years, recurrent/refractory medulloblastoma, AT/RT, or ependymoma	NK cell harvest, expansion, release, and safety of 112 intraventricular infusions of NK cells were achieved without dose-limiting toxicity. Eight patients had progressive disease and one showed stable disease.	^12^
NCT01875601	NK White Blood Cells and Interleukin in Children and Young Adults with Advanced Solid Tumors	I	16	Ages 2–29 with refractory pediatric malignant solid tumors	Results not yet available	–
NCT02100891	Phase II STIR Trial: Haploidentical Transplant and Donor Natural Killer cells for Solid Tumors (STIR)	II	15	All ages, solid tumors, and CNS tumors (high-risk malignant brain tumors that are recurrent/refractory)	Donor NK infusions were well-tolerated without cytokine release syndrome. Median follow-up was 1.3 years with 1 and 2 yr OS of 64% and 40% respectively for the entire cohort. Disease control rate at 6 mo was 72%.	^114^
NCT01213407	Dendritic Cell Cancer Vaccine for High-Grade Glioma	II	87	Ages 3–70, Supratentorial glioblastoma at the time of diagnosis with a total, subtotal, or partial resection of more than 70% by MRI	Progression-free survival at 12 mo did not differ significantly between control and vaccine group. Median overall survival was similar with 564 days in the vaccine group and 568 in the control group.	^118^
NCT02130869	A Pilot Study of Immunotherapy Including Haploidentical NK Cell Infusion Following CD133 + Positively Selected Autologous Hematopoietic Stem Cells in Children With High Risk Solid Tumors and Lymphomas	I	8	Age <22 years, Group C includes high-risk brain tumors	Results not yet available	-
NCT04214730	Study of NK Combined with Chemotherapy for Advanced Solid Tumor		60	Ages 10–90, patients with treatment refractory advanced solid cancer that are non-operable	Study ongoing	-

### Medulloblastoma

Extensive genomic, epigenomic, and transcriptomic research efforts have led to the identification of four molecularly distinct subgroups in medulloblastoma. These are not only characterized by distinct biological features but also linked to different clinical outcomes. Various studies have further defined substantial intertumoral heterogeneities within each of the molecular subgroups^122^. Diverse immune-stromal patterns involving macrophages, T cell-associated mechanisms, and NK cell function, results in distinct mechanisms of immunosuppression in various molecular subgroups of medulloblastoma^123,124^. This reaffirms the need to better understand the medulloblastoma tumor microenvironment interplay, which could help in improving therapeutic outcomes of NK cell therapy targeting this neoplasm.

Earlier studies have shown some clinical efficacy and safety, using lymphokine-activated killer cells (LAK cells) that were administered intrathecally^125^. Medulloblastoma express specific ligands that trigger NK activating receptors (commonest being the NKG2D), and thus are susceptible to NK-mediated cytotoxicity. Major histocompatibility complex class I-related chain A (MICA), CD1d and UL16 binding protein 2 (ULBP-2) are cell surface ligands of medulloblastoma, that bind NK cell activating receptor NKG2D. These interactions favor developing NK cell-based therapeutics against these tumors^62,126^.

TGF-β is a potent mechanism for immune invasion and its pro-migratory potential induces metastasis in medulloblastoma^127^. Group 3 with c-MYC amplification is the most virulent and formidable subtype of medulloblastoma. TGFβ signaling is upregulated by the exopolyphosphatase PRUNE 1 inducing metastatic medulloblastoma^128^. Engineered cord blood-derived NK cells expressing a TGFβ dominant-negative receptor-2 (TGF-β DNRII), significantly undermined the inhibitory effects of the tumor microenvironment induced by TGFβ. This efficacy could now be pursued as a mode of targeted therapy in these tumors^72^. The recently completed first-in-human trial with intraventricular infusions of NK cells in pediatric malignant brain tumors, including medulloblastoma, has demonstrated safety and some transient responses^12^.

The immune checkpoint B7-H3 is expressed in nearly 95% of medulloblastoma^129,130^. This has motivated the development of novel B7-H3 directed immunotherapeutic methods for refractory or recurrent medulloblastoma. Recent preclinical data showed successful development of a tri-specific killer engager platform (TriKE^TM^), assembled by DNA shuffling and ligation using DNA encoding a camelid anti-CD16 antibody fragment, a wild-type IL-15 moiety and an anti-B7-H3 scFV. These TriKE activated and targeted NK cells to inhibit the growth of various B7-H3-positive human cancer cells. This promising data provides an opportunity for this molecule to target B7-H3 in medulloblastoma^131^. A radioimmunotherapy Phase I/II trial of intracerebroventricular infusion of 177Lu-DTPA-omburtamab (antibody to B7-H3) was initiated in recurrent medulloblastoma. Preclinical data showed NK cell migration continued into tumors, as these infusions were given, suggesting an immune benefit (NCT04167618).

### Pediatric high-grade glioma

Pediatric high-grade glioma (pHGG) comprise approximately 11% of all pediatric brain tumors^132^. Despite multimodal therapy; including surgery, systemic chemotherapy and irradiation; the 5-year overall survival for recurrent pHGG is dismal^133^. Lack of sustainable efficacious modalities has led to innovative research to improve these outcomes. The tumor microenvironment, particularly the immune microenvironment, is being examined for possible therapeutic implications.

Historically, NK cell function within pHGG has been limited by the immunosuppressive landscape of these tumors^7^. Factors such as PD-L1, B7-H3, IL-8, and TGFβ restrict the brain’s anti-tumor response, furthering the tumor’s aggressive nature^134–137^. TGFβ and IL-8 are readily expressed in pHGG, and directly reduce NK cell activating receptors; as well as antagonize IL-15, which increases NK cell proliferation, activation and decreases apoptosis^138,139^. In glioblastoma there is evidence suggesting that these tumors have increased expression of CD99, which is induced by TGFβ, inferring higher TGF-β in the tumor microenvironment^68^. Furthermore, pre-clinical data demonstrates that NK cells retrovirally transduced to express TGF-β-dominant-negative receptor II (DNRII) are efficacious in evading TGF-β mediated mechanisms of NK cell suppression, making them an attractive therapeutic modality^140–142^.

For NK cell therapy to be efficacious in these highly aggressive tumors, it is imperative to mitigate the cytotoxic activity TGF-β exerts on adoptive immunity^143–145^. A preclinical study showed that treatment of glioblastoma stem cell-engrafted mice, with allogeneic NK cells in combination with inhibitors of integrin or TGF-β signaling or with TGFBR2 gene-edited allogeneic NK cells, prevented GSC-induced NK cell dysfunction and tumor growth. This phenomenon should now be explored in clinical trials^73^.

Additional novel immune therapies, such as oncolytic viral therapy and CAR-NK therapy, are also being explored in these tumors. Oncolytic viral therapy induces tumor killing, both through direct cytotoxic selective viral replication in cancer cells as well as through immune recruitment. In fact, studies have demonstrated that immunosuppressive cytokine expression decreases oncolytic viral efficacy and overall survival, while immunostimulatory expression improves survival in pre-clinical glioma models^146,147^. Recently, Jennings et al examined the use of histone deacetylase (HDAC) inhibitor priming to overcome the immunoregulatory impact of these factors on oncolytic viral therapy. In this study the HDAC inhibitor, valproic acid, augmented the expression of activating NK ligands on human melanoma cells, and when co-cultured with a herpes simplex oncolytic virus, NK-mediated tumor lysis was increased^148^.

Based on its utility and efficacy in melanoma, oncolytic viral therapy is now being translated to early phase clinical trials in adults with high-grade glioma^149^, though it remains in infancy in the pediatric neuro-oncology world. A recently completed oncolytic HSV-1G207 immunotherapy for pediatric HGG has been completed in 12 patients. Radiographic or clinical responses was seen in 11 patient’s and there was no dose-limiting toxicity. G207 infusions converted this immunological cold formidable tumor to a hot phenotype. Furthermore, significant infiltration of lymphocytes including NK cells into the tumor were observed^13^.

Elimination of glioblastoma by NK cells, was markedly enhanced through expression of chimeric antigen receptor (CARs), targeting relevant antigens such as ErbB2 (HER2). These genetically engineered cells enhance efficacy, not only by directly killing tumor cells, but also by augmenting tumor killing through crosstalk with dendritic cells^150^. Encouraging pre-clinical data of CAR-NK cells in glioblastoma (as well as solid tumors) have led to two adult clinical trials (NCT04489420, NCT03383978)^105,150,151^. These trials however are limited to adult subjects, once again demonstrating the need for pediatric-specific clinical trials.

## Challenges facing NK cell therapeutics in brain tumors

NK cell therapy in brain tumors is emerging as a promising tool, due to their superior safety profile, absence of graft-versus-host disease, cytokine storm and neurotoxicity compared to T cell-based therapies^152^. Nevertheless, NK cell therapies are hindered by some limitations. NK cells comprise 5–15% of circulating lymphocytes in adult humans^153^. The cost of treatment and the difficulties to meet optimal clinical-grade ex vivo expansion have posed challenges to the advancement of NK cell therapies^7,154^. CAR NK therapies use NK cells derived from peripheral or umbilical cord blood^155^; however, transfecting these NK cells with the CAR constructs is more difficult than T cells^156,157^. Gene editing NK cells using CRISPR Cas9 techniques can help overcome this problem^158^. The life span of NK cells with the CAR constructs may be shorter than that of CAR T cells^159^. Designing CAR NK cells that overexpress IL-15 and directed towards CD19 prolonged NK cell survival to at least 4 weeks in preclinical mouse models^86^. In human clinical trials, higher or multiple doses of NK cells could be needed to induce a meaningful therapeutic effect^160^. Tumor heterogeneity poses another limitation, as it becomes necessary to identify a universal tumor antigen/target for CAR NK cell constructs. Brain tumors, such as gliomas, can have decreased expression levels of proteins that inhibit NK cell activity^16,37^. The addition of CAR NK cells to the natural NK cells in circulation can lead to increased cytotoxicity^140,161^, and immune side effects. The BBB restricts the passage and trafficking of immune cells, such as NK cells, into the brain^8^. Intrinsic factors, such as transcription factors, and extrinsic factors, such as integrins, selectins, cytokines, and chemokines, in the cell control trafficking and homing of NK cells to the tumor microenvironment^162^. Immune cell infiltration varies in patients with brain tumors^163–165^; higher infiltration is associated with better survival in glioma^37^. Local delivery of NK cell therapeutics via intraventricular and/or intrathecal routes can aide with tumor coverage and NK cell distribution.

## Imaging tools to understand homing of therapeutic NK cells

In parallel to the surge of NK cell therapeutics, recent years have seen a constant improvement of molecular imaging techniques, to evaluate the tracking of infused NK cells. These include quantitative dynamic footprinting (qDF) and total internal reflection fluorescence (TIRF) microscopy^166–168^, Förster resonance energy transfer (FRET) imaging^169–171^, optical live-cell imaging (including multiphoton and confocal imaging)^172–174^, light-sheet microscopy^175,176^, and super-resolution microscopy^177–179^. These techniques facilitate the visualization of immune responses at subcellular and molecular level in real time. This will facilitate profiling optimal and effective NK cell therapies^180^. Conventional cytological and histopathological methods usually rely on chemical fixation and are limited in the resolution of dynamic molecular and cellular processes. However, NK cell delivery, homing, and trafficking to and within the tumor are dynamic, time-dependent contributory factors. Monitoring of the ongoing differentiation of transferred NK cells as well as their persistence and activity in the tumor are of particular interest to facilitate therapy assessment and profile clinical optimization (Table 5).

**Table 5:** Imaging of NK cells in brain tumor therapy.

Imaging modality		Preclinical application		Clinical application	Advantages	Disadvantages	Ref
		In vitro	In vivo				
Optical imaging	BLI	+	+	−	• Fast acquisition • High temporal resolution • Inexpensive • Sensitive • No radiation exposure	• Immunogenic • Limited tissue penetration • Poor spatial resolution	^188,191–193^
	FLI	+	+	−	• Fast acquisition • High temporal resolution • Inexpensive • Easy/diverse dye labeling • No radiation exposure	• Immunogenic • Limited tissue penetration • Poor spatial resolution	^189–191,193,197,217–219^
Nuclear imaging	PET	−	+	+	• Sensitive • High tissue penetration • Detailed spatial resolution • Accurate quantitation	• Slow acquisition • Expensive • Intricate labeling	^186,196,198^
	SPECT	−	+	+	• Sensitive • High tissue penetration • Accurate quantitation • Long-term monitoring • Cheaper than PET	• Slow acquisition • Expensive • Intricate labeling • Restricted spatial resolution	^186,197,198,219^
MRI		−	+	+	• High-resolution anatomical and molecular information • No radiation exposure	• Slow acquisition • Expensive • Low sensitivity • Contrast leak from NK cells • Iron oxide labeling decreases cell viability	^71,200,203,204,206,220–223^

*BLI* bioluminescence imaging, *FLI* fluorescence lifetime imaging, *PET* positron emission tomography, *SPECT* single-photon emission computerized tomography, *MRI* magnetic resonance imaging.

Intense interrogation of the molecular interactions between natural killer cells and the target cells, have unfolded the functional heterogeneity and subclasses of NK cell behavior, as they migrate into the tumor cells. This dynamic data has allowed categorization of NK cells into 5 distinct classes as they migrate towards the target cells, following their infusion^181,182^. Thus, imaging technologies of NK cell-based brain tumor therapies are being investigated to understand the real-time spatiotemporal distribution and concentration of transferred NK cells to and within tumor sites.

Various methods have been developed in recent years for the noninvasive in vivo tracking of NK cells by molecular imaging^183^. These technologies can provide visualization and allow for characterization as well as quantification of biological, pathophysiological processes involved in NK cell-based treatments^184^. Future efforts should continue to improve NK cell molecular imaging strategies, to improve understanding of in the in vivo kinetics (trafficking, persistence) of NK cells to the tumor, potential therapeutic efficacy, off-target effects, and dynamics of the immunosuppressive microenvironment^184^. To date, these methods are restricted to preclinical models of brain tumors. The stage is now being set for the gradual translation of in vitro to in vivo settings in animals and humans to facilitate an in-depth exploration of NK cell behavior in the context of brain tumor biology and specific microenvironments^185^, with the most promising strategies being summarized in the following section of this review.

A growing body of noninvasive imaging modalities are currently being investigated to track immune cells, including optical imaging using fluorescence lifetime imaging (FLI), bioluminescent imaging (BLI), radionuclide imaging using single photon emission computed tomography (SPECT), positron emission tomography (PET) and magnetic resonance imaging (MRI)^186^. Many of these techniques rely on directly labeling the NK cell surface with fluorophores or loading of NK cells with contrast agents, radioisotopes, or cell-permeable fluorophores to allow for real-time visualization of NK cell-based immunotherapies^187,188^.

Direct or indirect labeling of cells are potential ways to track therapeutic NK cells via optical imaging. For example, NK cells can be directly labeled with exogenous fluorescent tracers for FLI. Fluorescent dyes commonly used in FLI include endogenous fluorophores, such as phenylalanine, NAD(P)H and flavin mononucleotide, organic dyes, fluorescent proteins, fullerenes, and quantum dots, all of which are applicable to permanently label NK cells through covalent and/or noncovalent binding to DNA, RNA, and proteins, reactions in the cytosol, and cell membrane insertion^189,190^. Indirect NK cell labeling is performed by transfection of reporter genes that induce formation of a protein product detectable via BLI (e.g., luciferase) or via FLI (e.g., green fluorescent protein)^191,192^. These NK cell labeling methods exhibit high sensitivity and specificity in small animal models, including mice and rats. Optical imaging has demonstrated a very high signal-to-noise ratio, the potential of providing multiplex imaging using various probes with diverse spectral characteristics and is accompanied with low instrumentation expenses^193^. Although appealing and widely used in the preclinical field, optical imaging is challenged by the small depth of tissue penetration (1 mm in FLI, 3 mm in BLI), restricted spatial resolution (2–3 mm) and poor anatomical discrimination, which limits its use to small animal studies and precludes its evaluation in larger animals and humans^188^.

The two nuclear imaging modalities used to track immune cells (PET and SPECT) are well integrated in the clinic and can be applied to visualize NK cell dynamics in animals and humans due to their capability of visualizing in vivo cell migration anywhere in living organisms and providing three-dimensional imaging data. PET and SPECT have the advantages of high sensitivity and unlimited depth penetration, excellent signal-to-background ratios, and a broad range of clinically applicable probes, many of which are FDA-approved^194,195^. From a technological perspective, these modalities are limited by its poor spatial resolution of approximately 1 to 5 mm, restricted anatomical information, and rapid decay of radionuclide tracers (e.g., 2 to 4 h for 18FDG), which limits the number of scanning sessions. Furthermore, these imaging modalities are associated with high cost and patients are exposed to ionizing radiation, which presents a concern^196,197^. Computed tomography can be used to improve PET and SPECT resolution, through attenuation correction and allows for the correlation of tracer signal with anatomical structures^198^.

MRI is well-established in the clinical practice and readily translatable from preclinical to clinical applications. This imaging modality offers high-resolution anatomical information good depth of penetration in tissue, well-defined soft tissue contrast and specificity, and lacks exposure to ionizing radiation. Constraints of MRI compared to optical imaging and radiotracer-based imaging modalities are the low sensitivity for molecular detection, including small NK cell populations, long scan times, and relatively high costs^199^. In vivo quantitation of NK cells is feasible using relaxation rate maps. However, this is an indirect quantification approach, as the detected MRI signal has to be computationally related to perturbations in tissue proton magnetization rather than directly to cell concentration^188^. 19F MRI is another MRI-based cell tracking technique that has received increasing attention in recent years. This technology builds upon the natural abundance of the tracer and offers high MRI sensitivity and background free images. 19F MRI, which is a promising quantitative molecular imaging approach due to the high sensitivity and low endogenous background signal of the 19F atom in vivo, has recently been approved by the FDA after several studies showed its potential for tracking NK cell-based immunotherapies^200,201^. 19F perfluorocarbon has been successfully used in preclinical models of medulloblastoma to demonstrate intracranial delivery of therapeutic NK cells. 19F MRI provided adequate imaging feedback of 19F-labeled NK cells, which distributed throughout the target area. Importantly, 19F-labeling did not undermine NK cell-cytotoxicity^71^ and suppressed medulloblastoma growth. A separate study supported these findings, demonstrating that NK cell labeling with the 19F isotope caused no detrimental effect on cellular function as indicated by maintained cytotoxicity of leukemia cells^202^.

Nanoparticles, such as superparamagnetic iron oxide and ultra-small superparamagnetic iron oxide nanoparticles, have also been used to label NK cells for MRI^203,204^. Loading NK cells with iron oxide nanoparticle is relatively easy and involves simple incubation, electroporation, or use of a transfection agent^205^. Iron oxide-labeled NK cells produce strong hypointense signals in T2 and T2* weighted images, and depending on how long the adoptively transferred NK cells survive, the iron oxide label can be retained for up to 4 days^206^. Several iron oxide nanoparticles are FDA-approved and in clinical use (ferumoxide, ferumoxytol, and ferucarbotran), underscoring the promising translation potential of paramagnetic particles-labeled NK cells. Nevertheless, the accurate interpretation of iron oxide nanoparticle-induced loss in MRI signal intensity remains a challenging task since other factors also contribute to hypointensity on T2 and T2* images, such as susceptibility artifacts due to air–tissue interfaces, magnetic field inhomogeneities due to poor shimming, presence of blood, and others.

Although each of the discussed imaging modalities individually is saddled with limitations, which impede their development into a gold standard for quantitatively monitoring in vivo distribution and timing of NK cell trafficking, multimodal hybrid imaging of two or more imaging technologies holds promise for future clinical applications. Specifically, MRI and PET, or optical imaging and MRI, offers the most desirable characteristics for tracking NK cells qualitatively and quantitatively, measuring cellular viability through visualization of signal colocalization, and assessing therapeutic response to NK cell adoptive therapy. The last few years have yielded promising results in the expansion, labeling, and in vivo tracking of NK cells^199^. Future research efforts should focus on refined, optimal, and robust imaging modalities to precisely quantify the homing, bio-distribution, and persistence of adoptively transferred NK cells in humans with an intent to improve clinical outcome^188^.

## Conclusion and future perspectives

Despite technological advances in the multimodal treatments for patients with brain tumors, significant relapses and treatment-led toxicities continue to undermine therapeutic efficacy. The last decade has witnessed a surge in cellular therapy for these formidable neoplasms. As an alternative to T cell therapy, use of NK and CAR-NK cell therapeutics are gaining momentum in solid tumors, due to the absence of graft-versus-host disease, potential to generate “off-the-shelf” products with NK cell lines, iPSC-NK or expanded NK cells from blood, umbilical cord blood or other hematopoietic progenitors. The immediate availability of product is critically important in aggressive CNS tumors where therapy cannot wait for production delays. Continuing efforts should also focus on strategies using loco-regional infusions of NK cells (intra-tumoral and intraventricular), enhancing its tumor delivery by overcoming blood–brain barrier, minimizing risk of rejection, and undermining the suboptimal systemic delivery of cellular therapy into these CNS tumors. The future should also see significant advances in labeling and in vivo tracking of NK cells to tumor sites in real time, to optimize clinical efficacy of NK cell-based therapeutics in neuro-oncology.
